# Trends in Japanese Spotted Fever Outbreaks in Fukuyama City of Hiroshima Prefecture in Japan, a High-incidence Area

**DOI:** 10.31662/jmaj.2025-0013

**Published:** 2025-08-08

**Authors:** Masahiro Okada, Kazumasa Okada, Kengo Banshoya, Yasumasa Yamamoto, Shinya Okamoto, Fumiyoshi Murakami, Kazuko Okazaki, Narumi Sugihara

**Affiliations:** 1 Department of Pharmacy Onomichi Municipal Hospital, Hiroshima, Japan; 2 Myoodai Elementary School, Hiroshima, Japan; 3 Faculty of Pharmacy and Pharmaceutical Sciences, Fukuyama University, Hiroshima, Japan; 4 Kume Internal Medicine Clinic, Ehime, Japan

**Keywords:** Japanese spotted fever, Fukuyama City, high-incidence area, temperature

## Introduction

Japanese spotted fever (JSF), a tick-borne rickettsiosis transmitted by the bite of ticks harboring *Rickettsia japonica*, is endemic to Japan. The characteristic clinical features of JSF include fever, rash, and eschar ^(1)^. The first cases of JSF were reported by Mahara et al. ^(1)^ in 1984, and have increased in recent years ^(2)^. Tetracycline is the first-line treatment for JSF ^(1)^. Kutsuna et al. ^(3)^ reported that patients who received tetracycline on the second day of hospitalization or later had higher in-hospital mortality, longer hospital stays, and higher hospitalization costs than those who received tetracycline on the day of hospitalization ^(3)^. Therefore, physicians must diagnose JSF accurately, quickly, and promptly to initiate tetracycline therapy in patients with suspected JSF ^(3)^.

Fukuyama City, located in the eastern part of Hiroshima Prefecture, is a high-incidence area for JSF ^(4)^. However, there are no reports on the incidence of JSF in Fukuyama City and the factors associated with it. In this report, we describe the incidence of JSF in Fukuyama City and the factors associated with its onset with the aim of contributing to its early diagnosis and treatment.

## Methods

First, we investigated the number of JSF cases in Fukuyama City and nationwide from 2020 to 2023 and calculated the number of JSF cases per 100 000 individuals. Second, we investigated the factors associated with the occurrence of JSF. We selected temperature and precipitation as the factors associated with the occurrence of JSF. Data were taken from the Fukuyama City Infectious Disease Surveillance ^(5)^ and National Institute of Infectious Diseases, Infectious Diseases Weekly Report for 2020-2023 ^(6)^, and the 2020 population census of Japan ^(7)^. JSF is a notifiable disease classified as a category IV infectious disease. The number of JSF cases in Fukuyama City used data reported to Fukuyama City Public Health Center. Diagnosis of JSF relied on administrative tests such as serodiagnosis, using polymerase chain reaction (PCR) for blood or crusts followed by direct sequencing of PCR products. Information on temperature and precipitation was obtained from the Japan Meteorological Agency ^(8)^; monthly mean temperature (Celsius) and monthly total precipitation (mm) in Fukuyama City were used in this study. The optimal cut-off values for temperature and precipitation were calculated on the basis of the receiver operating characteristic curve analysis. Statistical analyses were performed using logistic regression. The presence or absence of JSF by month of onset in each year was defined as the dependent variable, and temperature and precipitation were defined as independent variables. Statistical significance was set at *P* < 0.05. Statistical analyses were performed using EZR version 1.40 (Saitama Medical Center, Jichi Medical University, Saitama, Japan) ^(9)^.

## Results

The number of JSF cases in Fukuyama City from 2020 to 2023 was 93, with an incidence rate of 5.04 cases per 100,000 individuals per year. During the same period, the number of cases in Japan was 1870, with an incidence rate of 0.37 cases per 100,000 individuals per year; therefore, the incidence rate of JSF cases in Fukuyama City was 14 times that of the national average.

**Figure 1.**
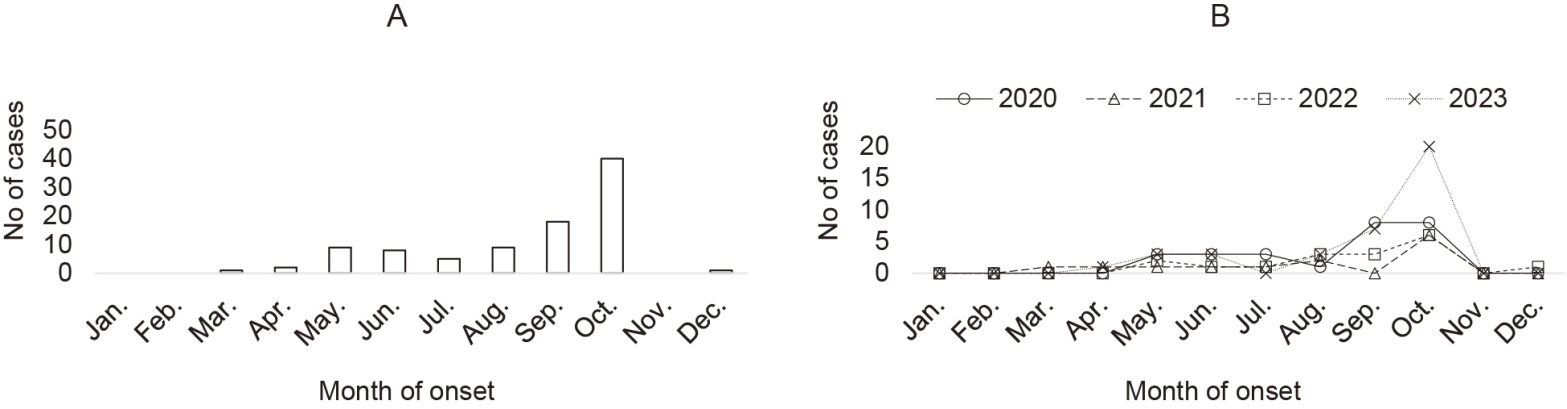
Number of cases in 2020-2023 (A) and in each year (B) of Japanese spotted fever in Fukuyama City by month of onset. The number of cases per month was bimodal, with a small first peak in May and a larger second peak in October.

JSF cases in Fukuyama City occurred from early summer to autumn, and the monthly number of cases was bimodal, with a small first peak in May and a larger second peak in October (Figure 1).

Table 1 lists the results of the association assessment between the onset of JSF in Fukuyama City and temperature or precipitation in Fukuyama City. The onset of JSF in Fukuyama City was associated with temperatures above 14.3ºC (odds ratio [OR] = 73.70, 95% confidence interval [Cl]: 6.59-826.00, p < 0.01). The onset of JSF in Fukuyama City was not associated with precipitation above 83.5 mm (OR =1.05, 95 % confidence interval [Cl]: 0.10-11.30, p = 0.97).

**Table 1.** Factors Associated with the Onset of Japanese Spotted Fever in Fukuyama City.

Parameter		OR (95% Cl)	p Value
Temperature (Celsius)	<14.3	Reference	
	≥14.3	73.70 (6.59-826.00)	<0.01
Precipitation (mm)	<83.5	Reference	
	≥83.5	1.05 (0.10-11.30)	0.97

Cl: confidence interval; OR: odds ratio

## Discussion

The incidence of JSF continues to increase in Japan ^(1)^, especially in the eastern prefectures, where JSF has not been previously diagnosed ^(10)^. In addition, many cases of JSF in Japan may be underdiagnosed because a considerable percentage of patients do not notice the rashes and eschar ^(11)^. Therefore, attention should be paid to the occurrence of JSF, not only in Fukuyama City but also in all prefectures in eastern Japan.

The incidence of JSF per 100,000 individuals from 2020 to 2023 was 5.04 cases/year in Fukuyama City and 0.37 cases/year nationally. The incidence of JSF in Fukuyama City was 14 times higher than the national average. This study focused on Fukuyama City, an area with a high incidence of JSF. We also investigated the incidence of JSF in Fukuyama City and the factors associated with its onset.

JSF is generally recognized as an infectious disease that occurs from spring to autumn ^(12)^. Fukunaga reported that the incidence of JSF increases from May to October and peaks from August to October, coinciding with the tick activity season ^(13)^. This study also showed that JSF cases occurred from early summer to autumn, which is consistent with the findings of previous studies. Furthermore, we observed that the monthly number of JSF cases showed a bimodal pattern, with a small first peak in May and a larger second peak in October. Animals, including humans, are bitten by ticks because adult and nymphal ticks are active from spring to early summer and larvae are active in autumn ^(14)^. The small first peak in May may be due to bites by adult and nymphal ticks, and the larger second peak in October may be due to bites by larval ticks.

We selected temperature and precipitation as factors associated with the occurrence of JSF based on a study by Ogawa et al. ^(2)^. In this study, the onset of JSF was associated with temperature above 14.3°C. Ogawa et al. reported that the regional incidence of hospitalization for JSF negatively correlated with low temperature ^(2)^. They also reported that cases of JSF were concentrated in the western part of the country, which may be due to environmental factors such as temperature that affect tick survival in these regions ^(2)^.

This study had several limitations. First, there is a possibility of selection bias because this study was only investigated in Fukuyama City, an area with a high incidence of JSF. Second, because JSF is transmitted by the bite of ticks carrying *R. japonica*, the carriage rate of *R. japonica* may vary depending on the tick species and habitat ^(15)^. Therefore, it is necessary to clarify the details of the carriage rate of *R. japonica* by tick species and habitat, in addition to environmental factors such as temperature. According to the study by Shimazu et al. ^(4)^, JSF is similarly common in the surrounding areas of Fukuyama City. It is possible that the approach to JSF (number of doctors diagnosing or awareness activities) may differ between Fukuyama City and other areas and prefectures in Hiroshima Prefecture, apart from in terms of tick species, habitats, and the prevalence of *R. japonica*. Moreover, because Fukuyama City is an area with a high incidence of JSF, it is possible that testing for suspected cases of Japanese myiasis is more proactive in Fukuyama City.

In conclusion, the results of this study on the incidence of JSF in Fukuyama City, number of cases per month, and factors associated with the incidence of JSF can be useful when JSF is suspected as a potential diagnosis.

## Article Information

### Conflicts of Interest

None

### Acknowledgement

We thank Saki Okada for assisting with this study and Editage (www.editage.jp) for English language editing.

### Author Contributions

Masahiro Okada: Conceptualization, Methods, Investigation, Formal analysis, Writing―original draft; Kazumasa Okada: Conceptualization, Investigation, Formal analysis; Kengo Banshoya: Methods, Formal analysis, Writing―review and editing; Yasumasa Yamamoto: Writing―review and editing, Supervision; Shinya Okamoto: Writing―review and editing; Fumiyoshi Murakami: Writing―review and editing; Kazuko Okazaki: Writing―review and editing, Project administration; and Narumi Sugihara: Writing―review and editing, Project administration. All authors gave their final approval for the version to be published and agreed to be accountable for all aspects of this report.

### Approval by Institutional Review Board (IRB)

Ethical approval was not required because this study was conducted using surveillance data. No identifiable information has been reported in this study. This study complies with the Declaration of Helsinki and was performed.
